# The peripheral blood transcriptome reflects variations in immunity traits in swine: towards the identification of biomarkers

**DOI:** 10.1186/1471-2164-14-894

**Published:** 2013-12-17

**Authors:** Núria Mach, Yu Gao, Gaëtan Lemonnier, Jérôme Lecardonnel, Isabelle P Oswald, Jordi Estellé, Claire Rogel-Gaillard

**Affiliations:** 1 INRA, UMR1313 Génétique Animale et Biologie Intégrative, F-78350 Jouy-en-Josas, France; 2 AgroParisTech, UMR1313 Génétique Animale et Biologie Intégrative, F-78350 Jouy-en-Josas, France; 3 Department of Nutritional Sciences, University of Wisconsin-Madison, Madison, USA; 4 INRA, UMR1331, Toxalim, Research Centre in Food Toxicology, F-31027 Toulouse, France; 5 Université de Toulouse III, INP, Toxalim, F- 31076 Toulouse, France

**Keywords:** Biomarkers, Blood, Pig, Transcriptome, Immune response

## Abstract

**Background:**

Immune traits (ITs) are potentially relevant criteria to characterize an individual’s immune response. Our aim was to investigate whether the peripheral blood transcriptome can provide a significant and comprehensive view of IT variations in pig.

**Results:**

Sixty-day-old Large White pigs classified as extreme for *in vitro* production of IL2, IL10, IFNγ and TNFα, phagocytosis activity, *in vivo* CD4^-^/CD8^+^ or TCRγδ + cell counts, and anti-*Mycoplasma* antibody levels were chosen to perform a blood transcriptome analysis with a porcine generic array enriched with immunity-related genes. Differentially expressed (DE) genes for *in vitro* production of IL2 and IL10, phagocytosis activity and CD4^-^/CD8^+^ cell counts were identified. Gene set enrichment analysis revealed a significant over-representation of immune response functions. To validate the microarray-based results, a subset of DE genes was confirmed by RT-qPCR. An independent set of 74 animals was used to validate the covariation between gene expression levels and ITs. Five potential gene biomarkers were found for prediction of IL2 (*RALGDS*), phagocytosis (*ALOX12)* or CD4^-^/CD8^+^ cell count (*GNLY, KLRG1* and *CX3CR1)*. On average, these biomarkers performed with a sensitivity of 79% and a specificity of 86%.

**Conclusions:**

Our results confirmed that gene expression profiling in blood represents a relevant molecular phenotype to refine ITs in pig and to identify potential biomarkers that can provide new insights into immune response analysis.

## Background

Over the last decades, production traits have been studied and exploited for the genetic improvement of livestock through selective breeding [1]. At the same time, diseases that can cause substantial economic losses have emerged. There is little doubt about the economic and welfare implications of infectious diseases or about the existence of genetic variation in disease susceptibility and resistance in livestock populations [2]. However, research aimed at identifying genetic factors that confer relative susceptibility or resistance against diseases in pigs is limited by the lack of sufficient phenotypic observations. Thus, including health traits in the current breeding schemes while limiting loss in long-term selected production traits is a major concern and stands as an emerging trend in pig breeding [1,3-6].

Direct strategies that target animal resistance or tolerance to specific pathogens, may result in increased susceptibility to other diseases [7]. Therefore, we and other authors have suggested an indirect approach focused on immunity traits (ITs) [3,7,8]. ITs measured on healthy animals are studied as candidate traits for immune capacity. To define immune capacity or immunocompetence, it is necessary to know how the immune system of an individual responds to different stimuli (e.g. infection by microorganisms, vaccination or environmental stresses), and its efficiency. Therefore, the term immunocompetence may be defined as the ability of the host to launch an immune response of sufficient specificity and magnitude, and thus is a rough indication of the effective quality of the host’s immune system [9,10]. For instance, in poultry for which many studies are available, He *et al.*[11] demonstrated that chicken lines with functionally less active heterophils (equivalent to mammalian neutrophils) were more susceptible to infections by *Salmonella enteritidis* than those with highly functional heterophils. Furthermore, Swaggerty *et al*. [12] reported that broilers selected for higher levels of pro-inflammatory cytokines and chemokines had a more efficient pro-inflammatory profile that contributed in part to increased resistance against pathogens. Thus, identifying candidate ITs that could predict immunocompetence is a major issue in animal production systems. We and others have shown that a large number of ITs are heritable, which suggests that genetic selection on ITs is feasible [7,13]. The new challenge is to identify heritable ITs that are significantly associated with health or disease resistance and may predict the efficiency of an individual’s immune response before biotic or abiotic stresses occur [14].

Peripheral blood cells are now widely used as a surrogate tissue to monitor individuals for various markers [15]. Blood cells constitute one of the first lines of the immune defence system [16]. For studies on immunocompetence, blood is considered as a target tissue that contains the different immune cells that circulate in the whole body. Indeed, profiling gene transcripts in blood has earned its place in the molecular and cellular profiling approaches used to analyse the immune response in patients with a wide range of diseases in humans [17]. Moreover, analysis of the blood transcriptome can contribute to identify immune response specific signatures (overexpressed or under-expressed transcripts) associated with specific ITs, which might be translated into useful molecular biomarkers for differential immunocompetence. Ultimately these biomarkers or patterns of markers could help to improve animal selection programs.

Huang *et al.*[18,19] and Arceo *et al.*[19] have shown that pig blood transcriptome is informative to monitor disease susceptibility, to characterize response to immune stimulation [20] or to refine the characterization of certain ITs [8]. In the present study, our first objective was to perform blood transcriptome profiling in pigs with extreme IT levels and without any initial focus on resistance to specific pathogens. Our second objective was to investigate whether genome-wide transcriptional data in blood can lead to the identification of candidate biomarkers associated with variations of ITs.

## Results

### The blood transcriptome varies significantly for four of the eight ITs tested

Eight ITs were considered in an extreme phenotype study design (Table 1). The ITs were classified into two subsets corresponding to (1) ITs measured after *in vitro* stimulation (IL2, IL10, IFNγ, TNFα and phagocytosis capacity (PHAG)) and (2) ITs measured *in vivo* from blood (αβ T lymphocyte CD4^-^/CD8^+^ count (CD4^-^/CD8^+^), γδ T lymphocyte count (TCRγδ^+^), and level of IgG specific to *Mycoplasma hyopneumoniae* (IgG-Mh)). The average and standard deviation of extreme groups for each IT are in Table 1, and information on their distribution is in Additional file 1: Figure S1 and Additional file 2: Figure S2. On average, a statistically significant difference between the means of each pair of groups was observed for each IT at the 5% level (Table 1). We identified differentially expressed (DE) genes for IL2 and IL10 productions, PHAG and CD4^-^/CD8^+^ cell counts ITs (Table 2). Since gene expression was not significantly affected in the blood of pigs with different IFNγ, TNFα, TCR γδ^+^ counts and IgG-Mh levels, we focused our study on the association between IL2 and IL10 productions, PHAG and CD4^-^/CD8^+^ and gene expression. To validate technically the microarray gene expression data, blood RNA samples were analysed by real-time quantitative polymerase chain reaction (RT-qPCR) for 19 genes (Additional file 3). RT-qPCR results confirmed the microarray expression levels for 15 of the 19 selected genes (Additional file 4: Figure S3). Observed correlations between RT-qPCR results and microarray gene expressions were consistently high, with most genes having r^2^ values > 0.70.

**Table 1:** **Basic statistics describing the difference between high and low groups for *in vitro *** **production of IL2, IL10, IFNγ and TNFα, phagocytosis activity, and *in vivo *** **CD4**^**-**^**/CD8**^**+**^**and TCRγδ + cell counts, and anti-*Mycoplasma *** **antibody levels**

**Type of trait**	**Trait symbol**	**Group**	**Animal number**	**Mean**	**SD**^**5**^	**CV**^**6**^**(%)**	**p-value**
*In vitro stimulation*	IL2^1^	Low	11	-4.66	0.084	1.80	<0.0001
		High	10	3.20	0.243	7.59	
	IL10^1^	Low	10	-4.80	0.240	5.00	<0.0001
		High	10	1.71	0.056	3.27	
	IFNγ^1^	Low	11	-6.65	1.649	24.8	<0.0001
		High	7	2.63	0.174	6.06	
	TNFα^1^	Low	9	-2.41	0.496	20.61	<0.0001
		High	8	1.48	0.207	4.46	
	PHAG^2^	Low	11	4.53	0.326	7.06	<0.0001
		High	9	9.04	0.316	3.43	
*In vivo*	CD4^-^/CD8^+3^	Low	5	-3.52	0.697	19.80	0.0002
		High	3	2.96	0.622	7.43	
	TCR γδ^+3^	Low	8	-3.59	0.338	9.41	<0.0001
		High	9	4.49	0.338	7.52	
	IgG-Mh^4^	Low	10	-3.24	0.162	5.00	<0.0001
		High	10	4.12	0.776	18.83	

Traits were all transformed using Box-Cox transformation, thus making the transformed data symmetric; ^1^means were expressed as transformed pg of cytokine/mL of blood; ^2^phagocytosis activity was expressed as transformed percentages; ^3^leukocyte sub-populations were expressed as transformed cell counts/mL; ^4^total concentrations of immunoglobulin G against *M. hyopneumoniae* were expressed as transformed pg of IgG-Mh/mL of blood; ^5^standard deviation; ^6^coefficient of variation.

**Table 2:** **Differentially expressed genes for** ***in vitro*** **production of IL2, IL10, IFNγ and TNFα, phagocytosis activity,** ***in vivo*** **CD4**^**-**^**/CD8**^**+**^**and TCRγδ + cell counts, and anti-*Mycoplasma*** **antibody levels**

**Type of traits**	**Trait symbol**	**Number of animals**		**Number of differentially expressed genes**		
		**Low group**	**High group**	**Total**	**Up-expressed in High Group**	**Down-expressed in High group**
*In vitro*	IL2	11	10	850	413	437
	IL10	10	10	733	207	526
	IFNγ	11	7	0	0	0
	TNFα	9	8	0	0	0
	PHAG	11	9	1195	673	522
*In vivo*	CD4^-^/CD8^+^	5	3	52	10	42
	TCR γδ^+^	8	9	0	0	0
	IgG-Mh	10	10	0	0	0

### Differentially expressed genes in animals with contrasted *in vitro* production levels of IL2

**Figure 1:**
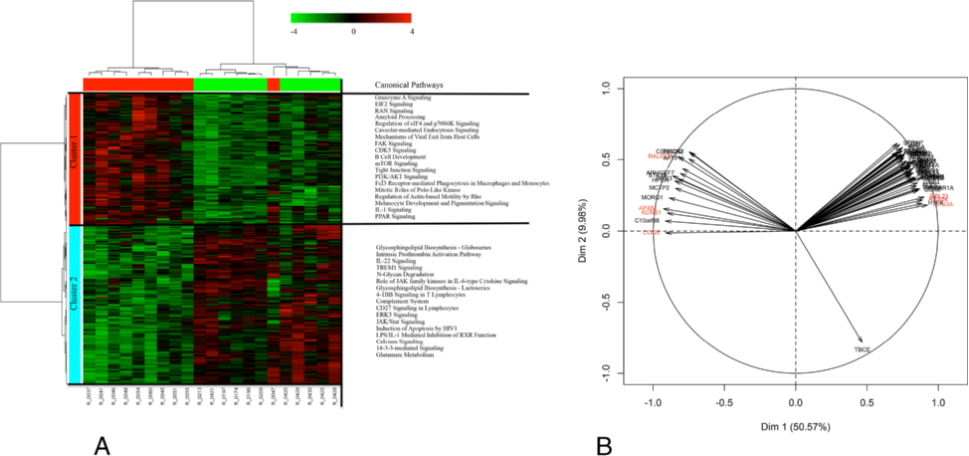
**Multivariate analyses of the differentially expressed genes in animals with contrasted IL10 production.** A two-way hierarchical clustering analysis matrix **(A)** and Principal Component Analysis gene factor map **(B)** are represented. In the heatmap, a color-coded gene module is displayed in the colour bars to the left of the dendogram. Each column in the heatmap corresponds to one animal (green for “Low group”; and red for “High group”). Furthermore, green colour represents low adjacency (negative correlation), while red represents high adjacency (positive correlation). In the PCA figure, the quality of representation of a variable on the axis is measured by the squared cosine between the vector issued from the element and its projection on the axis. The genes that contribute most to the separation between the two groups are coloured in red.

We identified 850 genes DE in the blood from pigs with extreme levels of IL2 (Table 2 and Additional file 5). The fold change (FC) of DE genes ranged from -2.67 to 2.62 when high (H) and low (L) groups were compared. Hierarchical cluster analysis (HCA) and principal component analysis (PCA) were applied to search for classifiers. The HCA animal dendogram separated the H and L groups, although one animal of the H group clustered with the piglets of the L group (Figure 1A). On the gene axis, two main gene clusters (clusters 1 and 2) were detected. A total of 413 genes in cluster 1 was over-expressed in animals of the H group compared to the L group (Figure 1A). Conversely, 437 genes in cluster 2 were significantly under-expressed in the H group versus the L group. The first component of PCA, projecting the arrows onto the first dimension, explained 50.57% of the total variability in gene expression and identified the DE genes that contributed most to the separation between the two groups (in red on Figure 1B).

**Figure 2:**
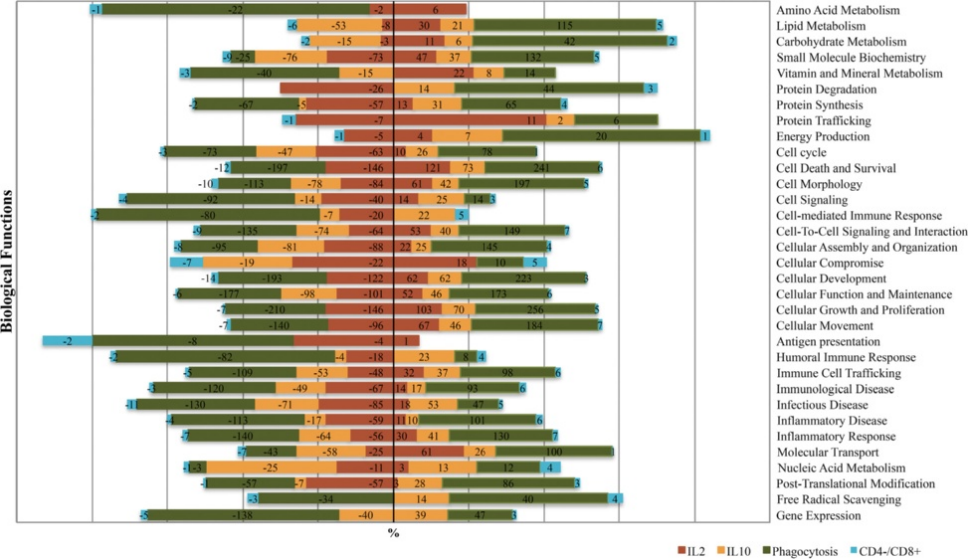
**Biological functions significantly affected when comparing the high versus low groups for*in vitro*production of IL2, IL10, phagocytosis activity and CD4**^**-**^**/CD8**^**+**^**cell counts.** Statistical significance of biological functions modulation was calculated via a right-tailed Fisher’s Exact test in Ingenuity Pathway and represented as –log (P value): -log values exceeding 1.30 were significant (FDR q-values < 0.05). For each pathway, the number of affected genes is indicated for each IT in the corresponding coloured box. The numbers of genes that were down-expressed (negative numbers) or up-expressed (positive numbers) in the high group are on the left and right sides of the graph, respectively.

To gain insight on the functions of the blood transcriptome that differed significantly between the H and L groups, we measured the subsets of DE genes by using the core analysis function included in Ingenuity Pathways Analysis (IPA). In the H group, IPA showed that the most significant over-expressed (*P* < 0.05) biological functions were related with cell growth, proliferation and development, cell death and survival, cellular and molecular transport, lipid metabolism, and immune cell trafficking (Figure 2, Additional file 6). By contrast, biological functions related to cell death and survival, cell signalling, cell-mediated immune response, immune cell trafficking, and inflammatory and infectious diseases were under-expressed (*P* < 0.05); Additional file 6). Significant canonical pathways that were induced in the H group compared to the L group were associated with mTOR signalling, PI3K signalling, regulation of eIF4 and p706K signalling and tight junctions signalling (Additional file 7).

### Differentially expressed genes in animals with contrasted *in vitro* production levels of IL10

**Figure 3:**
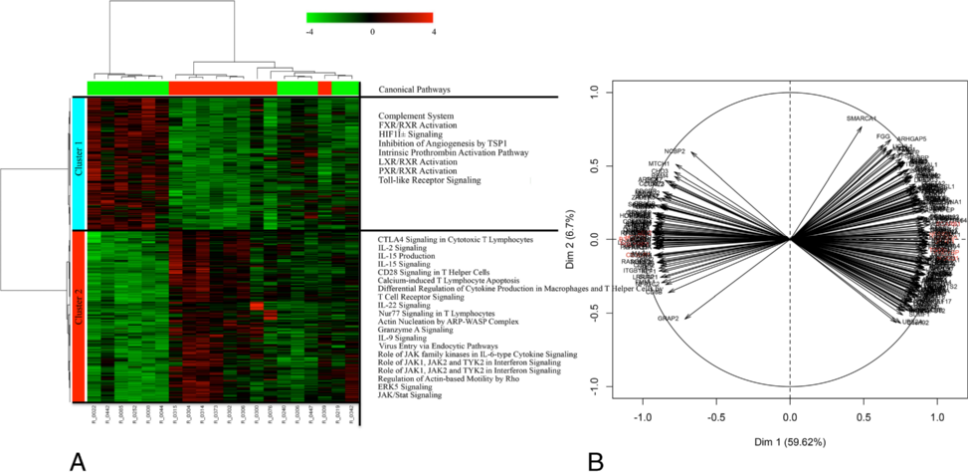
**Multivariate analysis of the differentially expressed genes in animals with contrasted IL10 production.** A two-way hierarchical clustering analysis matrix **(A)** and Principal Component Analysis gene factor map **(B)** are represented. In the heatmap, a color-coded gene module is displayed in the colour bars to the left of the dendogram. Each column in the heatmap corresponds to one animal (green for “Low group”; and red for “High group”). Furthermore, green colour represents low adjacency (negative correlation), while red represents high adjacency (positive correlation). In the PCA figure, the quality of representation of a variable on the axis is measured by the squared cosine between the vector issued from the element and its projection on the axis. The genes that contribute most to the separation between the two groups are coloured in red.

As shown in Table 2,733 genes were DE in the blood of pigs with contrasted IL10 levels (Additional file 8). The FC of the DE genes ranged from -2.65 to 1.93 when the H and L groups were compared. On the one hand, the HCA animal dendogram showed that one animal from the L group clustered within the H group (Figure 3A) and on the other hand, it identified two gene clusters: cluster 1 with 526 genes and cluster 2 with 207 genes. Most of the genes in cluster 1 were significantly down-expressed in the H group versus the L group, whereas in cluster 2 the opposite was observed (Figure 3A). In addition, the first component of PCA explained 58.75% of the total variability in gene expression (Figure 3B). In Figure 3B, the main genes that contribute to the separation of the two extreme groups are indicated in red. As shown in Figure 2, biological functions that were significantly less expressed (*P* < 0.05) in the H group compared to the L group were primarily involved in immune T-cell activation and trafficking functions (e.g., IL-2 signalling, IL-15 production and signalling, IL-17A signalling, and 4-1BB signalling in T cells), lipid metabolism, immunological diseases, cellular function, maintenance, assembly and organization (e.g., FAK signalling, HGF signalling, Rac signalling), as well as in cellular movement and proliferation (Figure 2, Additional file 6 and Additional file 9). By contrast, most biological functions associated with cellular movement, cell death and survival, and immune cell trafficking were over-expressed (*P* < 0.05) in the H group compared to the L group (Additional file 6 and Additional file 9).

### Differentially expressed genes in animals with contrasted phagocytic capacities

**Figure 4:**
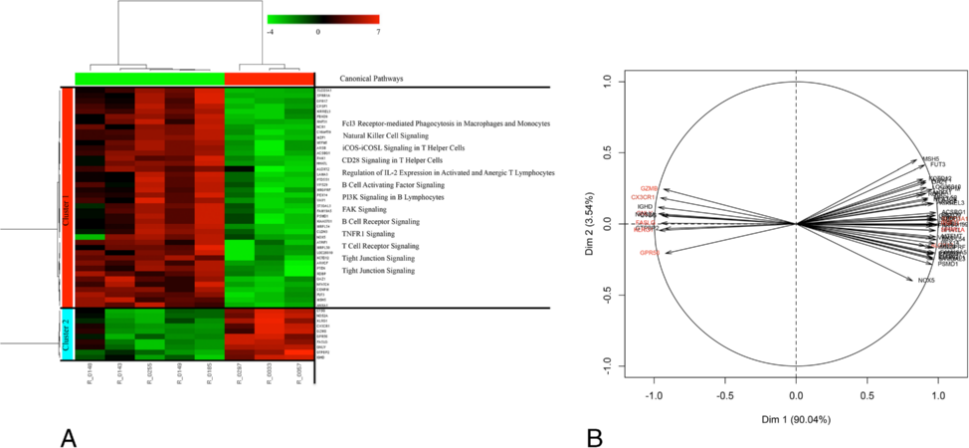
**Multivariate analysis of the differentially expressed genes in animals with contrasted phagocytosis activity.** A two-way hierarchical clustering analysis matrix **(A)** and Principal Component Analysis gene factor map **(B)** are represented. In the heatmap, color-coded gene module is displayed in the colour bars to the left of the dendogram. Each column in the heatmap corresponds to one animal (labelled by colour; Green for “Low group”; and Red for “High group”). Furthermore, green colour represents low adjacency (negative correlation), while red represents high adjacency (positive correlation). In the PCA figure, the quality of representation of a variable on the axis is measured by the squared cosine between the vector issued from the element and its projection on the axis. The genes contributing most to the separation between the two groups are coloured in red.

A large number of genes (1195) was significantly DE between animals with extreme phagocytic capacity (Table 2 and Additional file 10). The FC of the DE genes ranged from -3.49 to 2.51 between the two groups. Furthermore, the HCA animal dendogram separated two main clusters each with a mixture of animals belonging to both the H and L groups, which suggests that the L group could be split into two subgroups (Figure 4A). For the gene variables, two main HCA clusters were identified. Clusters 1 (522 genes) and 2 (673 genes) were primarily down-and over-expressed in the H group compared to the L group, respectively (Figure 4A). The first component of PCA (explaining 59.62% of the total variability) clearly separated the two groups and about 80% of the genes showed a high correlation with the two principal components (r^2^ > 0.7) (Figure 4B). Some of the most significant genes that separated the H and L groups are indicated in red in Figure 4B. Comparison of the extreme groups showed that the foremost over-represented IPA biological functions (*P* < 0.05) were related to nutrient metabolism (e.g. lipid metabolism, carbohydrate metabolism, protein degradation and trafficking, and energy production; Figure 2). Conversely, humoral immune response, infectious disease, cell-mediated immune response, cellular growth and proliferation, cellular maintenance and development, cell signalling, and amino acid metabolism were down-expressed (*P* < 0.05) in the H group versus the L group (Additional file 6). A precise examination of the canonical pathways revealed that inflammatory response, such as CD28 signalling in T Helper cells, CTL4A signalling in cytotoxic T lymphocytes, IL-2, IL-9, IL-15, IL-22 production and signalling, T cell receptor signalling, as well as JAK/Stat signalling were over-represented (*P* < 0.05) in the H group (Additional file 11). By contrast, in the L group, an increased expression (*P* < 0.05) of canonical pathways associated with acute phase response signalling, complement system, and LXR/RXR activation (Additional file 11) was observed.

### Differentially expressed genes in animals with contrasted αβ T lymphocyte CD4^-^/CD8^+^counts

**Figure 5:**
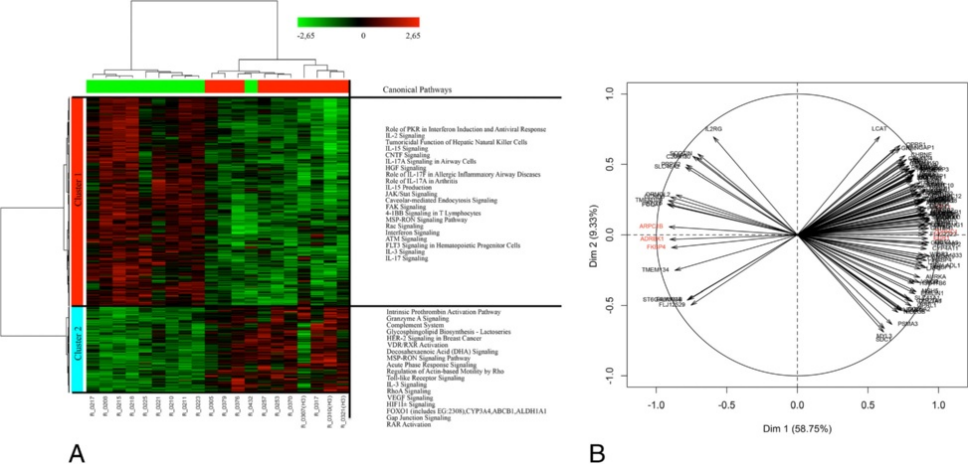
**Multivariate analyses of the differentially expressed genes in animals with contrasted CD4**^**-**^**/CD8**^**+**^**cell counts.** A two-way hierarchical clustering analysis matrix **(A)** and Principal Component Analysis gene factor map **(B)** are represented. In the heatmap, color-coded gene module is displayed in the colour bars to the left of the dendogram. Each column in the heatmap corresponds to one animal (labelled by colour; Green for “Low group”; and Red for “High group”). Furthermore, green colour represents low adjacency (negative correlation), while red represents high adjacency (positive correlation). In the PCA figure, the quality of representation of a variable on the axis is measured by the squared cosine between the vector issued from the element and its projection on the axis. The genes contributing most to the separation between the two groups are coloured in red.

Fifty-two DE genes were identified between groups with contrasted CD4-/CD8+ counts (Table 2; Additional file 12). The FC of DE genes ranged from -2.21 to 6.89 between the extreme groups. The HCA revealed a clear difference in gene expression patterns between the two groups. Indeed, the animal dendogram showed a higher divergence between individuals from different groups, which supports the fact that the small number of animals analysed per condition in this study was sufficient to reach a conclusion (Figure 5A). For the gene variables, two clusters were identified. Clusters 1 (42 genes) and 2 (10 genes) were respectively down- and up-expressed in the H group versus the L group (Figure 5A). The first component of the PCA captured 90.04% of the total variance, which indicated that this component represented most of the expression pattern of the individual samples. About 90% of the genes showed a high correlation with the two principal components (r^2^ > 0.7, Figure 5B). Genes that are highly correlated with the two principal components are indicated in red in Figure 5B. Regarding biological functions and canonical pathways, the DE genes were related to cell death and survival (18 genes), cell morphology (15 genes), cell-to-cell signalling and interaction (16 genes), cellular development (17 genes), and infectious diseases (16 genes; Figure 2; Additional file 6). However, the number of genes was too small for a reliable detection of enriched canonical pathways (Additional file 13).

### Identification of candidate biomarkers by testing a validation population

**Figure 6:**
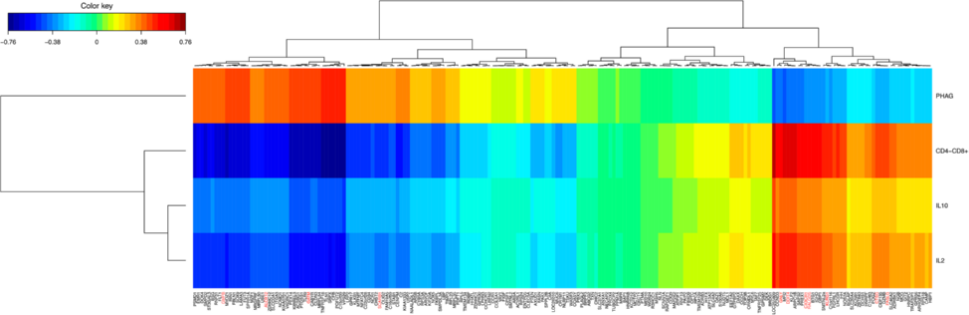
**Covariation between gene expression and levels of different ITs in a validation population using sparse partial least square regression.** The covariation between the blood transcriptome profiles of 74 animals and the corresponding levels of IL2 and IL10 production, PHAG activity and CD4^-^/CD8^+^ cell counts were measured. For each of these ITs, the top 50 differentially expressed genes between the high and low groups were selected. A total of 200 genes was included in the analysis. In the heatmap, increasing values are translated into colours from blue (negative association) to red (positive association). The symbol of the genes identified as candidate biomarkers is indicated in red.

**Figure 7:**
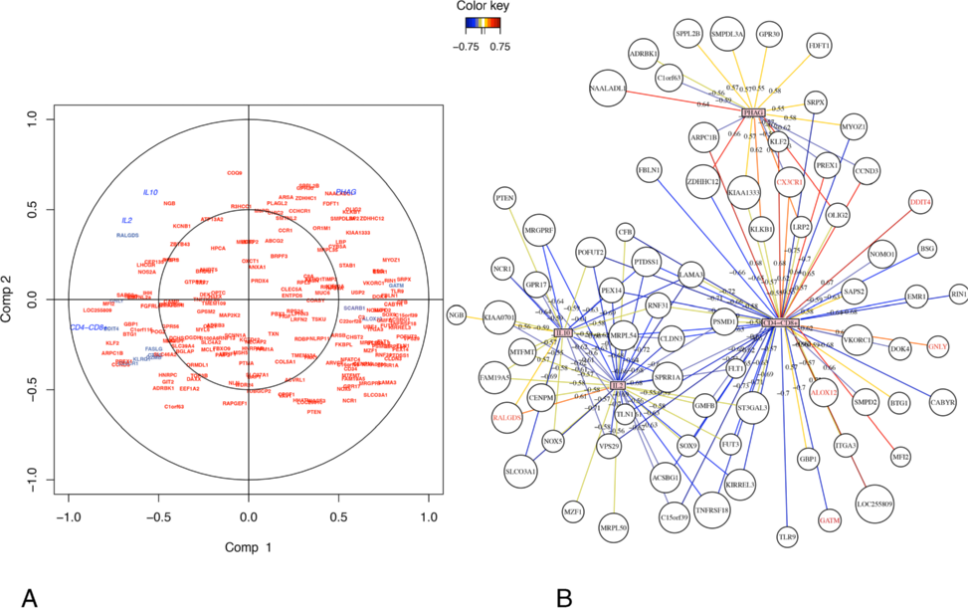
**Correlation between gene expression and levels of different ITs in a validation population using sparse canonical correlation.** The correlation between the blood transcriptome profiles of 74 animals and the corresponding levels of IL2 and IL10 production, PHAG activity and CD4^-^/CD8^+^ cell counts was measured. For each of these ITs, the top 50 genes that were differentially expressed between the high and low groups were selected. **A)** ITs and genes are represented through their projections onto a circle of radius 1 centred at the origin called correlation circle. Strongly associated variables are projected in the same direction from the origin. The greater the distance from the origin, the stronger the association. Only association scores greater than 0.50 with at least one of the ITs are displayed. **B)** ITs and genes are represented through a network. The network is displayed graphically as nodes (genes and ITs) and edges (the biological relationship between nodes). The edge colour intensity indicates the expression of the association: red = positive and blue = negative. Node shape indicates whether it is a gene (round) or an IT (square). The score of the association was indicated under each edge. Only pairwise associations with scores greater than 0.50 were projected. The symbol of genes identified as candidate biomarkers is indicated in blue in figure **A** and red in figure **B**.

**Figure 8:**
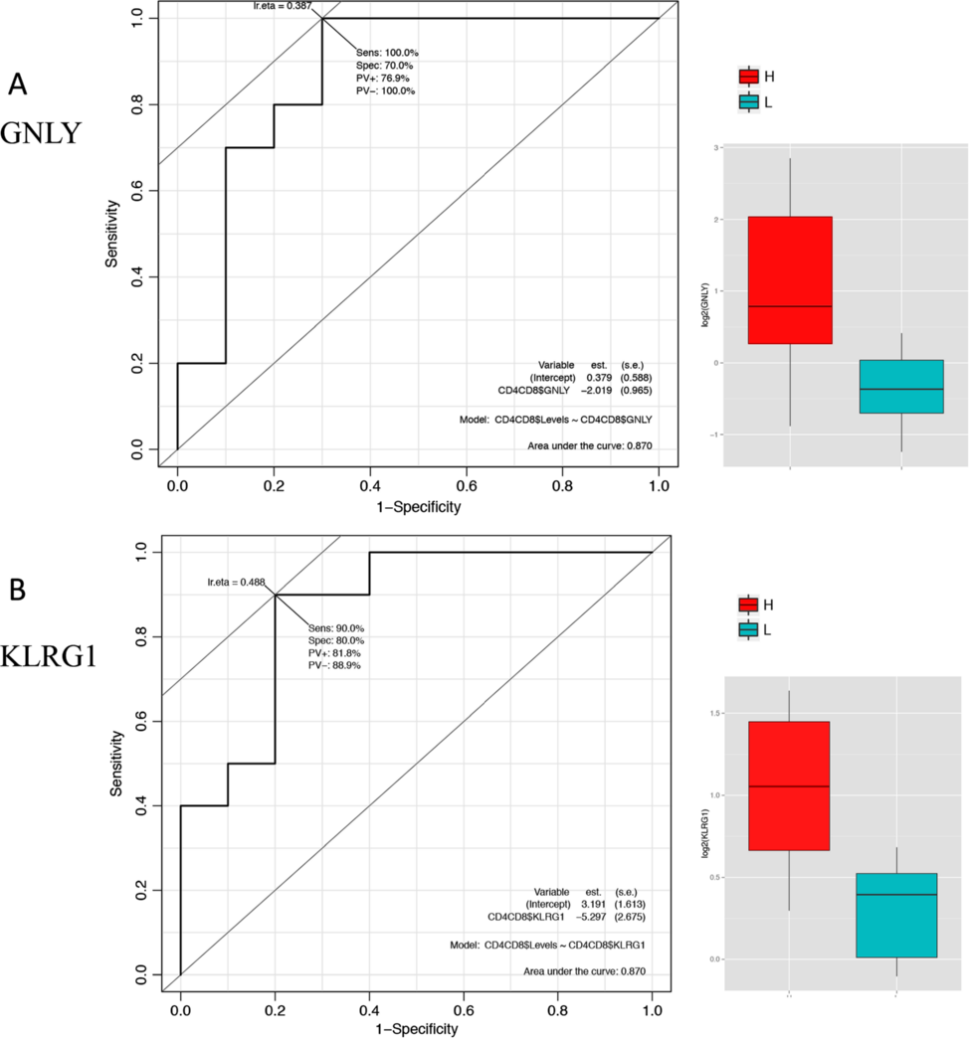
**Accuracy and reproducibility of*GNLY*(A) and*KLRG1*(B) genes using a discrimination analysis approach.** The receiver-operating characteristic (ROC) curve gave an area under the curve of 0.87 for both genes by comparing H and L groups for the CD4^-^/CD8^+^ cell counts. The solid black line represents the performance of the gene-expression biomarker on the test samples. The dashed line represents the line of no discrimination between H and L groups. The boxplot graph represents the expression levels (log2) of genes in the H and L groups for CD4^-^/CD8^+^ cell counts, respectively.

In order to validate our results and identify potential gene biomarkers for the ITs included in the study, we used a validation set of 74 animals. Sparse partial least square regression (sPLS) [21,22] and regularized canonical correlation analysis (rCCA) [23] statistical methods were applied to identify potential gene biomarkers. sPLS revealed a large covariance between expression of genes and distinct ITs. Q^2^ values, which have a meaning in terms of variable importance measure, showed that the best-explained variable was CD4^-^/CD8^+^ (Q^2^ = 0.134). Based on expression levels, most of the genes were negatively associated with IL2, IL10 and CD4^-^/CD8^+^ ITs, but positively associated with phagocytosis activity (Figure 6). The most striking result was the negative covariation between the following genes i.e. tumour necrosis factor receptor superfamily member 18 (*TNFRSF18*)*,* glycine amidinotransferase *(GATM),* mitochondrial ribosomal protein L54 (*MRPL54*)*,* arachidonate 12- lipoxygenase *(ALOX12),* complement factor B (*CFB)*, sushi-repeat containing protein *(SRPX),* protein O– fucosyltransferase 2 (*POFUT2*) or talin-1 (*TLN1*), and the IL2 and CD4^-^/CD8^+^ ITs. Another important finding was the strong positive covariation between DNA-damage-inducible transcript 4 (*DDIT4),* granulysin *(GNLY)*, the ral guanine nucleotide dissociation stimulator (*RALGDS)*, CX3C chemokine receptor 1 (*CX3CR1)*, Kruppel-like factor 2 (*KLF2*) and the CD4^-^/CD8^+^ IT. Focusing on correlation rather than covariance also enabled us to detect an association between the expression of selected genes and ITs (Figure 7). sPLS and rCCA analyses identified a total of 49 genes that were highly associated with at least one IT and that could be considered as potential gene biomarkers (similarity measure between a pair of vectors in the dimension 1 and 2 > 0.5). The suitability of these potential gene biomarkers was further assessed by the receiver operating characteristic (ROC) curve analysis and the area under the curve (AUC) in an extreme phenotype study design. Interestingly, the results demonstrated that 11 genes could be potential biomarkers to discriminate between H and L groups for IL2, PHAG and CD4^-^/CD8^+^ immune traits (Table 3). Among these 11 genes, *GNLY* and *KLRG1* achieved the highest predictive performance in discriminating high from low CD4^-^/CD8^+^ levels (Figure 8). The average expression intensity of the *GNLY* gene was 2.58 times greater in the H group than in the L group for CD4^-^/CD8^+^, with a sensitivity and specificity of 100% and 70%, respectively, and an AUC of ROC curve of 0.87 (Figure 8). The average expression intensity of *KLRG1* was two times greater in the H group than in the L group for CD4^-^/CD8^+^, with a sensitivity of 90% and a specificity of 80% and an AUC of 0.87. Lastly, the *CX3CR1* gene was also shown to be a good potential gene biomarker to differentiate between H and L groups for CD4^-^/CD8^+^. The *RALGDS* and *ALOX12* genes were identified as potential gene biomarkers to classify correctly IL2 production and phagocytosis activity, respectively (Table 3).

**Table 3:** Specificity, sensitivity and area under the curve of the 11 identified candidate biomarkers by testing a validation population

**Immune traits**	**Gene symbol**	**Parameters**		
		**Sensitivity (%)**	**Specificity (%)**	**AUC**^**1**^
IL2	*RALGDS*	70	100	0.84
PHAG	*GATM*	89	60	0.75
	*SCARB1*	88	50	0.68
	*ALOX12*	67	90	0.80
CD4^-^/CD8^+^	*GNLY*	100	70	0.87
	*FASLG*	70	70	0.67
	*DDIT4*	90	80	0.78
	*GZMB*	100	40	0.76
	*CTSG*	80	70	0.71
	*KLRG1*	90	80	0.87
	*CX3CR1*	70	90	0.82

^1^Area under the curve.

## Discussion

Blood transcriptome analyses were carried out to compare groups of animals with contrasted values for eight ITs. For each IT, we compared two groups of extreme animals, which presented significantly different mean values of the IT of interest. Because selection of the extreme animals included in the experimental sets was based on more stringent criteria and new quality filters were used in the gene analysis, the results reported here refine those of a previous report [8]. DE genes were identified for *in vitro* production of IL2 and IL10, phagocytosis activity and CD4^-^/CD8^+^ cell counts but not for the *in vitro* production of TNFα or IFNγ, the *in vivo* TCRγδ^+^ cell counts, and the levels of IgG-Mh.

### Blood transcriptome provides information to refine *in vitro* measured ITs: a potential of prediction

We identified gene expression profiles in blood (without stimulation) that were significantly associated with variations in ITs measured after *in vitro* blood stimulation, which suggests that the information provided by the peripheral blood can predict a response to a stimulus.

To our knowledge, the effects of varying IL2 levels on the genome-wide gene expression in the blood of pigs have not been studied to date. Although expression by naïve CD8^+^ T cells and dendritic cells has been reported [24], IL-2 is a cytokine produced primarily by activated CD4^+^ T cells. The main physiological functions of IL-2 are critical for the enhancement of cellular immune responses, and include induction of cytotoxic T cells, activation of natural killer cells (NK), production of other cytokines by T cells, stimulation of proliferation of activated B-lymphocytes, and induction of immunoglobulin secretion [25]. Here, we identified widespread changes in the expression of mRNAs in circulating blood cells that were obtained from animals with extreme levels of IL2 production after *in vitro* stimulation. The pathways involved in cell growth and proliferation were over-expressed in the H group, probably in accordance with a higher capacity to induce proliferation of B cells. Moreover, IL-2 is an important T-cell growth factor and appears to be required for naïve cells to develop into Th1 or Th2 cells [26].

Apart from TGF-β and IL-35, IL-10 is the most important cytokine with anti-inflammatory properties [27]. It is produced by almost all leukocyte types [28], and regulates the functions of many different immune cells: release of immune mediators, antigen presentation and phagocytosis [27]. IL-10 suppresses the functions of monocytes/macrophages that are responsible for both innate and specific immunities [27]. This agrees with our findings that animals with higher IL10 levels presented a decreased expression of biological functions related to cellular immune response, antigen presentation and inflammatory response. More specifically, we observed a reduction in IL2, IL-15, and IL-17A expression in animals with a high IL10 production in agreement with the results of Wolk *et al.*[28]. Since IL-10 is also involved in preventing apoptosis of B cells by enhancing their proliferation and differentiation [29,30], it was expected that growth and proliferation functions were over-expressed in animals with higher levels of IL10 compared to those with lower levels.

The blood transcriptome of animals with an extreme phagocytic *in vitro* activity revealed a large number of genes that were differentially expressed. One important observation of this study was that animals that have a higher *in vitro* phagocytic activity also have an activated CTL4A signalling pathway. This signalling pathway is necessary for the negative regulation of T-cell activity following T-cell activation by antigen-presenting cells [31]. Moreover, a strong relationship between blood phagocytosis and the stimulation of different interleukin pathways was detected, likely because cytokines are important mediators in the regulation of the immune and inflammatory responses. However, pathways associated with LXR/RXR activation were less expressed in animals with higher phagocytic activity. Interestingly, while the transcriptional pathways that allow macrophages to recognize and respond to apoptotic cells are poorly defined, Gonzalez *et al.*[32] reported that LXR signalling was important for both apoptotic cell clearance and maintenance of immune tolerance.

### Blood transcriptome provides information to refine *in vivo* measured ITs

For animals with extreme CD4^-^/CD8^+^ cell counts*,* transcriptome differences were shown to be the best predictors for phenotype variations compared to other ITs. The 52 annotated DE genes captured around 90.04% of the total variance. Moreover, the most highly expressed genes in animals with different levels of CD4^-^/CD8^+^ were the perforin and cathepsin complex gene members (*GNLY*, *GZMB),* natural killer cell-related genes (*KLRG1, NCR1),* chemokine receptor gene (*CX3CR1),* cytokine ligands such as Fas ligand TNF superfamily (*FASLG),* as well as resolution of acute inflammation resolving exudates genes *(ALOX12)*. There is evidence that *GNLY* and *GZMB* are involved in the synthesis of granzymes [33,34]. Saini *et al.*[35] reported that *GNLY* and *GZMB* genes may play a role in the elimination of cells infected with viruses or other pathogens, tumour surveillance, and transplant rejection. *FASLG*, an IFN-stimulated gene, has also been associated with cell death/apoptosis of uninfected bystander cells, and attack/killing of infected cells in pigs that recovered from a pestivirus infection [36]. Moreover, Marcolino *et al.*[37] and Voehringer *et al.*[38] reported that the gene for KLRG1, which belongs to the family of inhibitory C-type lectins that are encoded in the NK gene complex, is expressed in healthy human peripheral blood CD4 and CD8 lymphocytes. Interestingly, in pigs, it has been demonstrated that effector and memory CD4^+^ T cells express chemokine receptors such as CX3CR1 [39]. Although the functions of these genes remain to be elucidated in pigs, our results suggest that the levels of different subsets of αβ T lymphocytes affect the expression of genes related to antigen presentation, phagocytosis and immunoregulation.

### Blood transcriptome as a source of gene biomarkers for IT variation

The power of transcriptomics to identify potential gene biomarkers was previously demonstrated in classical studies on cancer, in which the analysis of gene expression signatures of primary tumours [40-42] led to the identification of predictive outcome profiles [43,44]. Most current strategies for the discovery of biomarkers involve a ‘top-down’ approach in which predictive genes are first identified by empirical association with a clinical symptom and are then evaluated as potential biomarkers for decision making [15]. In our study, the evaluation of potential biomarkers combined empirical association of gene expression with ITs and additional validation samples to demonstrate the accuracy and the reproducibility of the classifiers, using a multivariable analysis and a discrimination analysis approach. Five genes (*GNLY, KLRG1*, *ALOX12*, *CX3CR1* and *RALGDS)* were among the most promising potential gene biomarkers (Table 3). *GNLY*, *KLRG1* and *CX3CR1* genes were identified as potential gene biomarkers for the prediction of αβ T lymphocyte (CD4^-^/CD8^+^) counts. In humans, it has been shown that *GNLY* expressed in peripheral blood mononuclear cells (PBMC) is a biomarker for childhood and adolescent tuberculosis [45] and for the diagnosis of serious bacterial infections [46]. CXCR3 is a highly selective chemokine receptor and surface marker for cytotoxic effector lymphocytes, and KLRG1 is a surface marker used to predict the potential of CD8 effector T cells to differentiate into memory cells [47,48]. Moreover, Sherhan *et al.*[49] reported that *ALOX12* is associated with the synthesis of eicosapentaenoic acid, an essential fatty acid that can be enzymatically converted into E-series resolvins during inflammation in mammals. Understanding the mechanistic relationship and the biological meaning of these blood potential gene biomarkers is essential for future research.

### Blood transcriptome in healthy individuals as a source of relevant information for the prediction of immune traits

In humans and animals, blood is extensively monitored to follow health state, disease infection, and antibody production. In pigs, the blood transcriptome revealed variations in gene expression profiles in animals that differ in *Salmonella* shedding levels [18], and during the kinetics of response to porcine reproductive and respiratory syndrome virus infection [19]. Analysis of the transcriptome of PBMCs has also revealed variations in gene expression profiles in response to *in vitro* stimulation by lipopolysaccharide (LPS) or phorbol myristate acetate (PMA)-ionomycin (Iono) [50], vaccination with tetanus toxoid [20] or in response to *in vitro* pseudorabies virus infection [51]. This study shows that, based on an experimental design that does not target a response to an infection or an immune stress, blood transcriptome profiling is a valid molecular approach to identify potential biomarkers and biological pathways related to the function of the immune system in healthy animals that harbour different levels of *in vivo* as well as *in vitro* ITs. Additional studies will be necessary to ascertain dynamic changes that occur over time. In the future, it will be interesting to connect our results with those of a recent analysis on human blood transcriptome, that aimed at investigating the variations in gene expression in the blood of individuals within a population to predict the susceptibility to various environmental and living conditions [52]. Indeed, healthy individuals were categorized into nine common clusters based on co-regulated transcripts in the blood [52]. Each cluster was enriched for gene ontology categories related to subclasses of blood and immune functions. Therefore, understanding how the blood transcriptome varies across the population, and not only in the extreme individuals of the population, and correlating this variability with specific immune functions, could be an emerging component of the prediction of immune responses in pigs. Since CD4^-^/CD8^+^ and phagocytosis ITs are highly heritable in pigs [13] and associated with the expression of different genes, it might be anticipated that a study that combines the levels of gene expression with genetic analyses could contribute to identify candidate genes underlying heritable immune response traits. More studies are necessary on the functional and biological validation of blood gene biomarkers in pigs in order to better understand their role in the immune system response. Integration of information from various sources (e.g. immunity traits, stress traits, performance, health data) should be a major trend in the future to better understand causalities and promote prediction capacity. Lastly, since pig is an important biomedical model [53], profiling the blood transcriptome could be highly relevant to understand the immune function in animals, but also in humans. Recently, Groenen *et al.*[54] highlighted the pig as a relevant biomedical model and Dawson *et al.*[55] underlined the importance of the domestic pig as a model for human immunology since the two species share many pathogens.

## Conclusions

Peripheral blood represents an attractive tissue source because it is easily sampled and because of its potential as a sentinel tissue to monitor immunocompetence. Our results demonstrate that the transcriptome of circulating blood cells varies between healthy pigs with extreme levels of *in vitro* production of IL2 and IL10, phagocytic activity and CD4^-^/CD8^+^cell counts. Furthermore, five transcriptional biomarkers were found to be good predictors for CD4^-^/CD8^+^ cell counts, IL2 production, or phagocytic activity. Therefore, new molecular strategies to phenotype immune traits in pigs could be launched based on blood genome-wide transcriptome or by targeting specific biomarkers.

## Methods

### Animals

Animals were selected from a population of 443 Large White pigs (castrated males) bred in a test farm (UE450, INRA, Le Rheu, France). All animals were vaccinated with a single dose of inactivated *Mycoplasma hyopneumoniae* (Stellamune, Pfizer Animal Health) at 36 days of age, and blood was sampled three weeks later. We used animals that had already been measured for a large range of ITs in a previous study [13]. We focused on eight ITs (Table 1), including five ITs measured after *in vitro* stimulation (IL2, IL10, IFNγ, TNFα and PHAG) and three *in vivo* ITs directly measured in the blood (CD4^-^/CD8^+^ and TCRγδ^+^ counts), and serum (level of IgG-Mh). The choice of the ITs was based on the following criteria: (1) ITs that qualify the innate immunity or the cell and humoral-mediated adaptive immunity; (2) ITs that were highly (h^2^ > 0.4) or moderately heritable (0.1 < h^2^ ≤ 0.4), respectively [13].

All protocols for IT phenotyping are fully detailed in Flori *et al*. [13]. Briefly, for the IL2, IL10 and IFNγ dosage, 1:5 diluted blood was stimulated with a mixture of PMA and Iono for 48 hours and the levels of cytokines released in the supernatants were measured using in-laboratory developed ELISA tests. For TNFα, 1:5 diluted blood was stimulated with a mixture of PMA, Iono and LPS for 24 hours and the cytokine levels were measured using a commercial ELISA kit (DuoSet ELISA development kits, R&D Systems, USA). Phagocytosis was measured from total blood using the Phagotest kit (ORPEGEN Pharma, Heidelberg, Germany). The CD4^-^/CD8^+^ and TCRγδ^+^ cell counts were quantified by Fluorescence-Activated Cell Sorting (FACS), using FACScan and CELLQuest software (Becton Dickinson, Oxford, UK). Levels of specific IgG directed against *Mycoplasma hyopneumoniae* were measured in pig serum as described by Tarany *et al.*[56]. Box-Cox transformation of IT values was performed to improve the normality of the distributions and equalize variances to meet assumptions [57], thus making the transformed data symmetric.

For each of the eight ITs included, we created a specific experimental set. For each experimental set, pigs were selected from the higher and lower 10% of the Gaussian distribution in order to generate two extreme groups (high and low referred to as H and L groups, respectively). Furthermore, within groups, animals that contributed to increase the coefficient of variation of each IT (> 25%) were removed (Table 1, Additional file 1: Figure S1 and Additional file 2: Figure S2). There was no overlapping of individuals between the eight experimental sets. The number of animals included in each experimental set is given in Table 1.

All experiments on animals were conducted in accordance with the French national regulations for humane care and use of animals in research. No ethics approval was required for the vaccination and the collection of blood samples under the regulations applying at the time of the experiments. Experiments were performed under the individual license numbers 77–01 assigned to people responsible for experiments in the test farm. The experimentation agreement number for the test farm at le Rheu was A35-240-7.

### RNA isolation and microarray workflow

Blood for transcriptome analysis and phenotyping of ITs was sampled at the same time on PAXgene Blood RNA tubes (PreAnalystiX, Qiagen, Germany), in order to have a direct correspondence between the measured ITs and the blood transcriptome for each animal. Total RNA was isolated using PAXgene Blood RNA Kit (Qiagen, Germany) according to the manufacturer’s instructions. The RNA purity and concentration were determined using a NanoDrop 1000 spectrophotometer (Thermo Scientific, USA) and RNA integrity was assessed using the Bioanalyzer 2100 (Agilent Technologies, USA). A reference RNA was designed by combining total RNAs extracted from 17 different tissues: *in vitro* stimulated PBMCs (PMA-Iono), spleen, lymph node, thymus, ileal Peyer’s patches, liver, kidney, lung, testis, epididymis, ovary, uterus, heart, brain, *longissimus dorsi*, skin, and three weeks foetuses.

We used a microarray enriched for immunity related genes (SLA-RI/NRSGAP8-13 K long oligo microarray) as described in Gao *et al.*[50]. The microarray platform (GPL7151) consisted in 17 070 porcine oligonucleotide probes, representing at least 10 010 unique genes. All microarrays included in our study were produced by the INRA facility CRB GADIE (http://crb-gadie.inra.fr).

For each animal, 5 μg of total RNA were reverse transcribed and directly labelled by Cy3, and 5 μg of the reference RNA were reverse transcribed and labelled by Cy5, using the ChipShot™ Direct Labeling System (Promega, USA). The labelled cDNAs were purified with the ChipShot™ Membrane Clean-Up System (Promega, USA). Microarrays were pre-hybridized at 50°C for 30 min in a pre-hybridization buffer (3.5X SSC, 0.1% SDS and 1% BSA). The slides were then washed twice in distilled water for 5 min at room temperature and dried by centrifugation. A total of 750 ng of each Cy3-labeled and Cy5-labeled cDNA targets were mixed and the volume of the mix was adjusted to 200 μl with ddH_2_O water. The labelled cDNA mix was denatured at 95°C for 2 min, and 200 μl of 2X hybridization buffer (Agilent Technologies, USA) were added. The resulting 400 μl mixes were then centrifuged and applied to each single microarray with a cover slip (Agilent Technologies, USA). Microarrays were incubated at 60°C in an Agilent DNA microarray hybridization oven for 17 h. The slides were washed in 2X SSC 0.1% SDS twice for 5 min, 0.5X SSC and 0.1% SDS once for 5 min, 0.2X SSC three times for 5 min, and dried by centrifugation. The slides were scanned using the Agilent G2565CA scanner. For each array, the corresponding .tiff image was analysed using the GenePix^™^ Pro software V6.0 (MDS Inc., Canada).

All microarray experimental data are MIAME compliant and have been deposited in Gene Expression Omnibus ((GEO), http://www.ncbi.nlm.nih.gov/geo/) with the accession number: GSE45196.

### Statistical analysis of microarray expression data

All microarray analyses, including pre-processing, normalization and statistical analysis were carried out using ‘Bioconductor’ packages in R programming language (version 2.15). The homogeneity of the background was systematically checked on each microarray by the boxplot and image plot procedures of the linear models for microarray data (‘Limma’ library; version 3.14.4). Furthermore, PCA was performed with ‘FactoMineR’ library (version 1.23) to detect if any particular array contributed largely to variability in the gene expression data, that is, whether it retained most of the information (Additional file 14: Figure S4 and Additional file 15: Figure S5). Finally, after examining the resulting diagnostic plots, we analysed a total of 141 microarrays (Table 2).

The Log2 median ratio values between Cy3/Cy5 were normalised on each individual array (ratio centred on zero) according to the hypothesis that on the whole gene expressions do not differ between two samples. The centring was performed by ‘Lowess fitness’ to take into account the intensity dependence of the fluorescence bias. Identification of DE genes between groups was done with the ‘Limma’ package. The *P*-values were corrected for multiple testing using a false discovery rate method (*q*-value < 0.05), which provides an estimate of the fraction of false discoveries among the significant terms.

Centred on significantly expressed genes, unsupervised analysis was done to visualize clusters of samples or genes based on their variance-covariance structure. Such an analysis helps to define coordinated regulation of similarly related genes and study fundamental and intrinsic differences at the level of transcription that are specific to the groups studied. Thus, a two-way hierarchical cluster analysis was performed using ‘hclust’ function with ‘1-cor (x)’ as distance and ‘ward’ as aggregation criterion. The ‘heatmap’ function was used to generate images. In addition, PCA was performed with ‘FactoMiner’ library to better identify which genes contribute most to the separation of expression patterns between H and L groups. The quality of representation of a variable on the PCA axis is measured by the squared cosine between the vector issued from the element and its projection on the axis. If this square cosine is close to one, it means that the element is well projected on the axis [58]. The quality of representation of a gene on a plane can be visualized by the distance between the projected variable onto the plane and the correlation circle (circle of radius 1). The list of DE genes for each IT was uploaded into IPA (IPA; ver. 5.5, Ingenuity Systems, Redwood City, CA) to identify relevant categories of molecular functions, cellular components and biological processes. Using this approach, we identified statistically overrepresented functional GO annotations, and determined their up- or down-expression, and group-specific transcriptional networks. All listed or reconstructed cellular pathways were derived from the expert annotated database that is provided by the Ingenuity Knowledge Base. The IPA annotations follow the GO annotation principle, but are based on a proprietary knowledge database of over 10^6^ protein-protein interactions. The IPA output included biological functions and signalling pathways with statistical assessment of the significance of their representation based on Fisher’s Exact test. IPA computed networks and ranked them according to a statistical likelihood approach [59]. Only the canonical pathways that presented a -log (*P*-value) exceeding 1.30 (FDR q-values < 0.05) were described in the respective additional files. For the canonical pathways, the ratio values (number of molecules in a given pathway that meets cut criteria, divided by total number of molecules that make up that pathway) were also presented.

### Validation of the transcriptome results by RT-qPCR on a subset of genes

To technically validate the data generated in the microarray study, quantitative RT-qPCR was carried out on selected candidate genes (Additional file 3). The genes were selected based on the following strategies: (1) genes with significant DE levels between the phenotypes of interest that spanned a dynamic range of at least log2 (ratio) > 0.485; (2) genes with a coefficient of determination greater than 0.8 with respect to the first principal component in the PCA; and (3) genes with biological interest (e.g. *SLA-1* and *IL10RA;* Additional file 3). PCR primers specific to these genes were designed using ABI Primer Express software version 2.0 (Applied Biosystems, USA) and designed with the melting temperatures of 58°C to 60°C and resulting products between 100 and 150 bp. Briefly, reverse transcription of 1 μg of the isolated total RNA was performed using the high capacity cDNA archive kit (Applied Biosystems, USA), according to the manufacturer’s protocol. Dilutions were used for qPCR with SYBR green Master Mix (Applied Biosystems, USA) in an ABI Prism 7900 HT (Applied Biosystems, USA). The cDNA samples were mixed with 1× SYBR Green Master Mix and the specific reverse and forward primers, in a final volume of 20 μl. For each sample and each gene, PCR runs were performed in duplicates. In order to quantify and normalise the expression data, we used the ΔΔCt method using the geometric mean Ct value from the β-2 microglobulin (*B2M*) and L32 ribosomal protein gene *(RPL32)* as the endogenous reference genes [60].

The set of genes chosen for confirmation by RT-qPCR was analysed using a linear effect model, including group (H or L) as a fixed effect. Differences were considered significant at *P* < 0.05. The correlation analysis between RT-qPCR and microarray expression data was performed using the ‘corr’ function of R.

### Determination of potential blood biomarkers for immunocompetence

The top 50 DE genes between the H and L groups for IL2, IL10 production, PHAG activity and CD4^-^/CD8^+^ cell counts were selected to identify potential blood biomarkers (n = 200) in a validation set. To evaluate the predictive value of these selected gene signatures from the experimental sets, first, we mapped the gene signatures in a validation set of 74 animals. The validation set was sampled from the same original population to avoid biological and technical sources of dataset variation. These 74 animals corresponded to (1) animals that showed no differential expression between the H and L groups for the levels of IFNγ, TNFα and γδ T lymphocyte counts and IgG-Mh (n = 72), and (2) two animals that contributed to increase the coefficient of variation for CD4^-^/CD8^+^ cell counts, and that had been removed from the experimental set for this IT. All animals included in the validation set were analysed with the same SLA-RI/NRSP8-13 K microarrays and normalised as described above.

Two different statistical methods were applied to quantify the association between gene expression and the different ITs and to detect which of these genes could be considered as potential gene biomarkers: (1) the sPLS and (2) the rCCA. sPLS maximised the covariance between two datasets by searching for linear combinations of the variables. Furthermore, it imposed sparsity within the context of partial least squares and thereby carried out dimension reduction and variable selection simultaneously [21,22]. To evaluate the statistical significance of covariation between the expression of genes and the distinct ITs, we performed the M-fold or leave-one-out cross-validation, estimating the mean squared error of prediction (MSEP), the R^2^ and the Q^2^ for each IT in the dataset. An X variable contributed significantly to the prediction if Q^2^ ≥ (1–0.95^2^) [61]. rCCA identified and quantified the correlation between two datasets and regularized the empirical covariance matrices of X and Y by adding a multiple to the identity matrix [23]. The ‘mixOmics’ library (version 4.1.4) in R was used to carry out sPLS and rCCA analyses [62,63]. We used the ‘cim’ function to plot the sPLS results and the ‘plotVar’ and ‘network’ functions to generate the images from rCCA. On the one hand, the ‘cim’ function plotted the association matrices for X and Y variables. Increasing values were translated into colours from blue (negative association) to red (positive association). On the other hand, the variable plot (‘plotVar’) made it possible to identify the structure of the correlation between the two sets of variables X and Y. On the graphic, two circumferences were plotted with radiuses 0.5 and 1 to reveal the most salient patterns in the ring defined between these two circumferences. Variables with a strong relation were projected in the same direction from the origin. The greater the distance from the origin, the stronger the relation [23]. The ‘network’ function calculated a similarity measure between X and Y variables in a pair-wise manner. The output was a graph in which each X-and Y-variable corresponds to a node and the edges included in the graph display the associations between the nodes. Before considering a gene as a potential biomarker, constraints were applied. First, candidate potential gene biomarkers were selected if they presented at least a similarity measure (in absolute value) between a pair of vectors in the dimensions 1 and 2 greater than 0.50. Second, potential gene biomarkers had to be expressed in 100% of the animals. Then, the diagnostic of accuracy of potential gene biomarkers was assessed by ROC curve analysis in an extreme phenotype study design. For each potential gene biomarker, animals that were at the extreme ends (10%) of the phenotype distribution for the target ITs in the validation set of 74 animals were considered. On average, the number of individuals sampled from each tail was equal to 10. The ROC curve analysis was chosen as a measure of the accuracy of gene biomarkers because it includes all possible cut points and shows the relationship between the sensitivity of a biomarker and its specificity [64]. The AUC was calculated with the maximum area under a ROC curve equal to 1.00. An AUC of 0.5 indicates no association between prediction and the true outcome, and a value of 1 indicates perfect association. The optimal cut-off point was the point on the ROC curve closest to (0,1). The ‘epi’ library (version 1.1.49) in R was used to plot the ROC curve and find the optimal cut point.

### Availability of supporting data

The datasets that support the results of this article are available in the GEO database under the dataset identifier provided in the text.

## Abbreviations

ADRBK1: Adrenergic β receptor kinase 1; ALOX12: Arachidonate 12- lipoxygenase; AP4B1: Adaptor-related protein complex 4 and beta 1 subunit; ARPC1B: Actin related protein 2/3 complex subunit 1B; AUC: Area under the curve; B2M: β-2 microglobulin; B3GALT4: Phagocytosis activities were β-1,3-galactosyltransferase polypeptide 4 PGLYRP3, peptidoglycan recognition protein 3; CACYBP: Calcyclin binding protein; CB: Complement factor B; CCR1: Chemokine C-C motif receptor 1; CD4-/CD8+: αβ T lymphocyte CD4^-^/CD8^+^ counts; CHIC2: Cysteine-rich hydrophobic domain 2; COQ9: Coenzyme Q9 homolog; CTL4A: Cytotoxic T-Lymphocyte antigen 4; CTSG: Protein coding capthepsin; CX3CR1: CX3C chemokine receptor 1; DDIT4: DNA-damage-inducible transcript 4; DE: Differentially expressed; DEK: Oncogene; EGR1: Early growth response protein 1; FASLG: Fas ligand (TNF superfamily, member 6); FC: Fold change; FDFT1: Farnesyl-diphosphatefarnesyltransferase 1; FKBP4: FK506 binding protein 4; GATM: Glycine amidinotransferase; GEO: Gene Expression Omnibus; GKAP1: G kinase anchoring protein 1; GMFB: Glia maturation factor; GNLY: Granulysin; GPR56: G protein-coupled receptor 56; GZMB: Granzyme B; H group: High group; HCA: Hierarchical cluster analysis; HDAC5: Histone deacetylase 5; HEATR5A: HEAT repeat containing 5A; IgG-Mh: Immunoglobulin G directed against *Mycoplasma hyopneumoniae*; IL10RA: IL10 receptor; Iono: Ionomycin; IPA: Ingenuity Pathway Analysis; IT: Immune trait; KCNB1: Shab subfamily potassium cannel; KLF2: Kruppel-like factor 2; KLRG1: Killer cell lectin-like receptor subfamily G member 1; L group: Low group; LPS: Lipopolysaccharide; M. hyopneumoniae: *Mycoplasma hyopneumoniae*; MCCD1: Mitochondrial coiled-coil domain 1; MFI2: Antigen P97; MRPL54: Mitochondrial ribosomal protein L54; NCR1: Natural cytotoxicity triggering receptor 1; NK: Natural killer cells; NOMO1: NODAL modulator 1; PBMC: Peripheral blood mononuclear cell; PCA: Principal component analysis; PHAG: Phagocytosis; PLAGL2: Pleiomorphic adenoma gene-like 2; PMA: Phorbol myristate acetate; POFUT2: Protein O– fucosyltransferase 2; RALGDS: Theral guanine nucleotide dissociation stimulator; RNF31: Ring finger protein 31; ROC: Receiver-operating characteristic; RPL23: Ribosomal protein L23; RPL24: Ribosomal protein L24; RPL32: L32 ribosomal protein gene; RPS3A: Ribosomal protein S3A; RT-qPCR: Real time quantitative polymerase chain reaction; SCARB1: Scavenger receptor class B member 1; rCCA: Regularized canonical correlation analysis; SLA-1: Awine leukocyte antigen 1; SLCO3A1: Solute carrier organic anion transporter family, member 3A1; sPLS: Sparse partial least square regression; SPRR1A: Small proline-rich protein 1A; SRPX: Sushi-repeat containing protein; TCRγδ+: γδ T lymphocyte count; TGFB1: Transforming growth β-factor; TLN1: Talin-1; TNFRSF18: Tumour necrosis factor receptor superfamily member 18; ZDHHC1: GHHC domain-containing cysteine -rich protein.

## Competing interests

The authors declare that they have no competing interests.

## Authors’ contributions

NM carried out the statistical analysis and drafted the manuscript. YG participated in the design of the transcriptome study, carried out the RNA isolation and labelling, microarray hybridization and scan, and preliminary statistical analysis. GL carried out the RT-qPCR analyses. JL participated in the RNA isolation and microarray hybridization. JE participated in the design and supervision of the statistical analysis and RT-qPCR experiments, and helped to draft the manuscript. IPO helped to draft the manuscript. CRG designed and coordinated the study, supervised research and data interpretation, and contributed to draft the manuscript. All authors read and approved the final manuscript.

## Supplementary Material

Additional file 1: Figure S1 Distribution of the individual’s values for *in vitro* immune traits. The values of animals from the high and low groups are labelled by colour: green “Low” and red “High”. Click here for file

Additional file 2: Figure S2 Distribution of the individual’s values for *in vivo* immune traits. The values of animals from the high and low groups are labelled by colour: green “Low” and red “High”. Click here for file

Additional file 3 Sequences of the primers used for the RT-qPCR. Click here for file

Additional file 4: Figure S3 Validation of the transcriptome results by RT-qPCR on a subset of genes. Click here for file

Additional file 5 **Differentially expressed genes in animals with contrasted *in vitro *** **production levels of IL2.** Click here for file

Additional file 6 **Significance of the biological functions detected in animals with contrasted levels of IL2 and IL10 production, PHAG activity and CD4**^**-**^**/CD8**^**+**^**cell counts.** Click here for file

Additional file 7 **Differentially canonical pathways in animals with contrasted *in vitro *** **production levels of IL2.** Click here for file

Additional file 8 **Differentially expressed genes in animals with contrasted *in vitro *** **production levels of IL10.** Click here for file

Additional file 9 **Differentially canonical pathways in animals with contrasted *in vitro *** **production levels of IL10.** Click here for file

Additional file 10 Differentially expressed genes in animals with contrasted phagocytosis capacities. Click here for file

Additional file 11 Differentially canonical pathways in animals with contrasted phagocytosis capacities. Click here for file

Additional file 12 **Differentially expressed genes in animals with contrasted CD4**^**-**^**/CD8**^**+**^**cell counts.** Click here for file

Additional file 13 **Differentially canonical pathways in animals with contrasted CD4**^**-**^**/CD8**^**+**^**cell counts.** Click here for file

Additional file 14: Figure S4 Principal component analysis (PCA) of microarray expression data for *in vitro* immune traits. PCA was performed with FactoMineR library (version 1.23) to detect if any particular array largely contributed to variability in the gene expression data, that is, retains most information. Animals are labelled by colour: green “Low”; and red “High”. Click here for file

Additional file 15: Figure S5 Principal component analysis (PCA) of microarray expression data for *in vivo* immune traits. PCA was performed with FactoMineR library (version 1.23) to detect if any particular array largely contributed to variability in the gene expression data, that is, retains most information. Animals are labelled by colour: green “Low”; and red “High”. Click here for file
