# Supplementary figures and images for: The peripheral blood transcriptome reflects variations in immunity traits in swine: towards the identification of biomarkers
Source: BMC Genomics. 2013 Dec 17;14:894. doi: 10.1186/1471-2164-14-894 (PMC3878494; doi:10.1186/1471-2164-14-894)

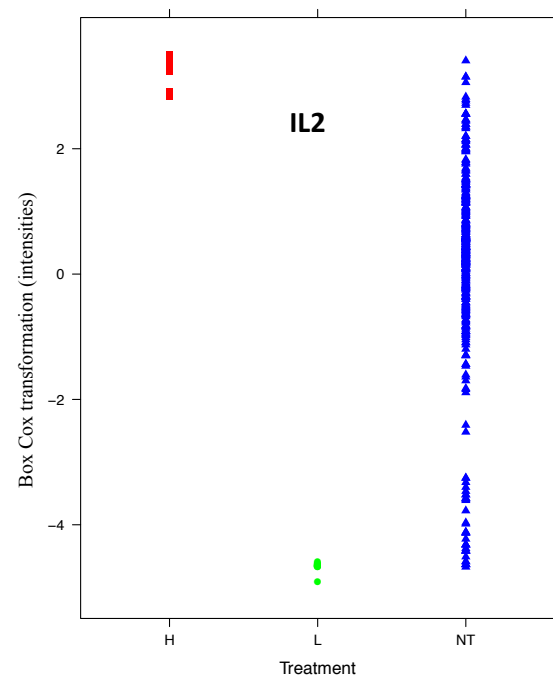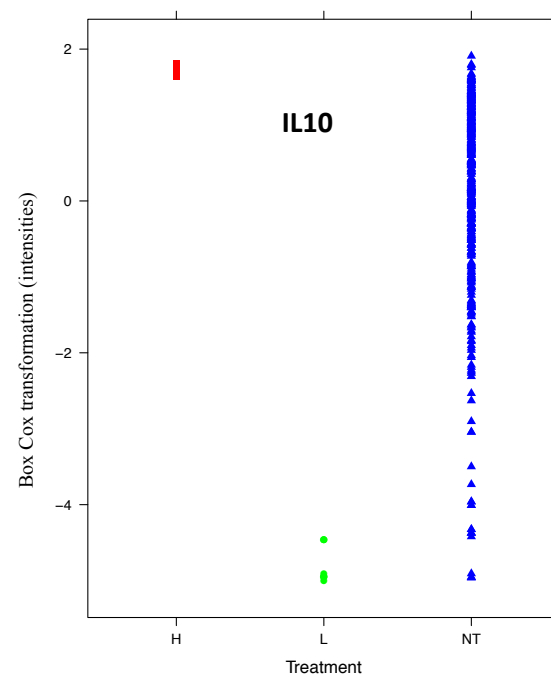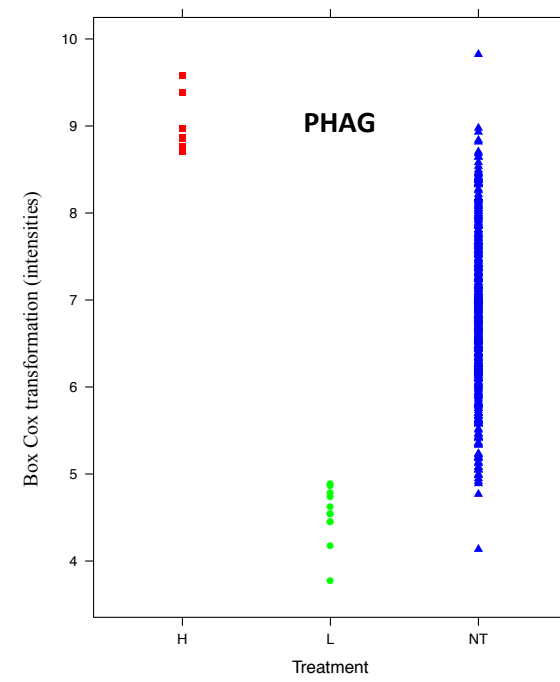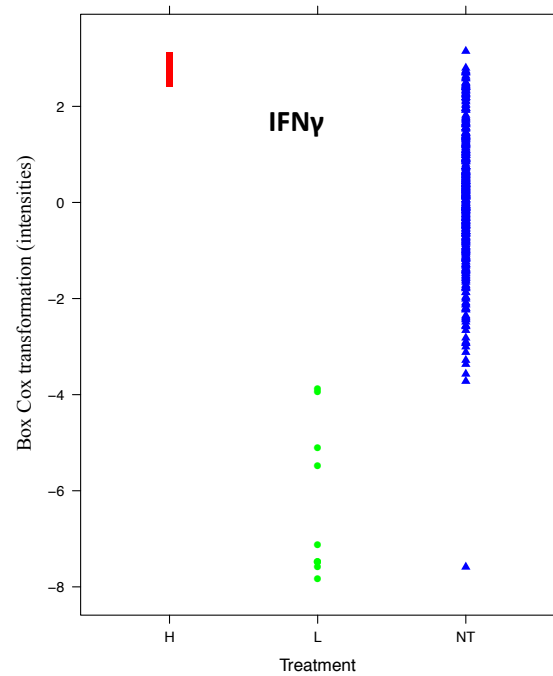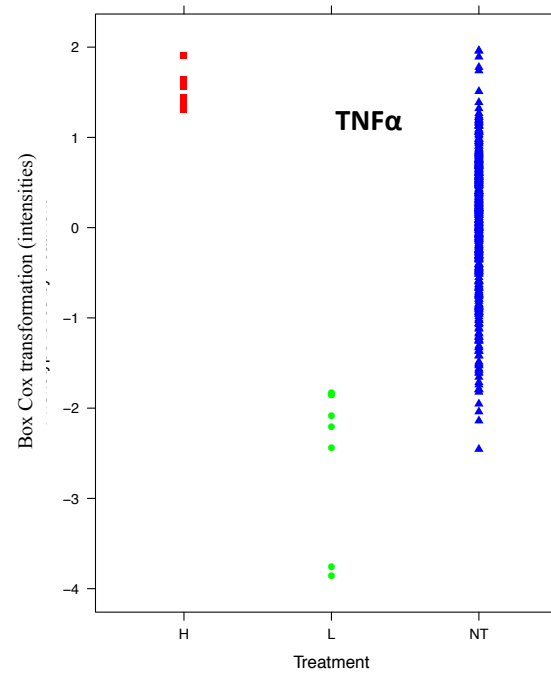

H ■ .....  
L ● .....  
NT ▲ .....  
.....

Supplement: Additional file 1: Figure S1 — Distribution of the individual’s values for in vitro immune traits. The values of animals from the high and low groups are labelled by colour: green “Low” and red “High”. [file 1471-2164-14-894-S1.pdf]

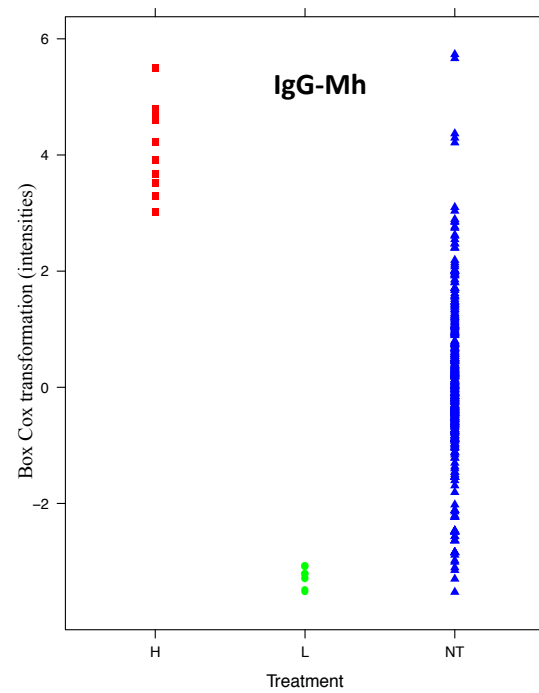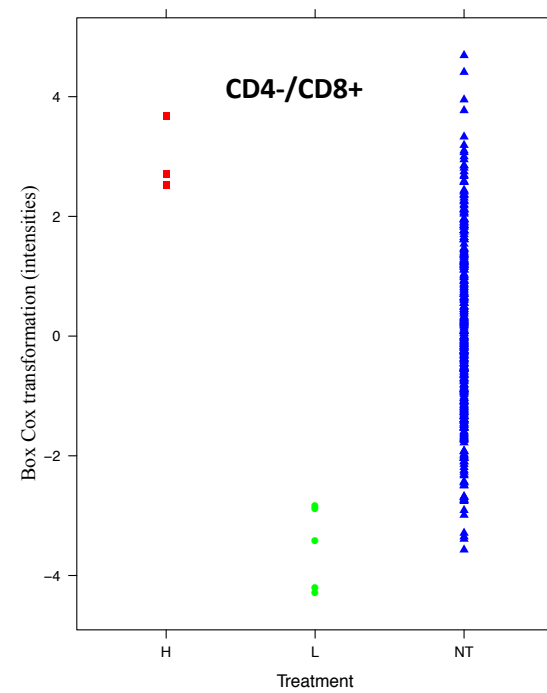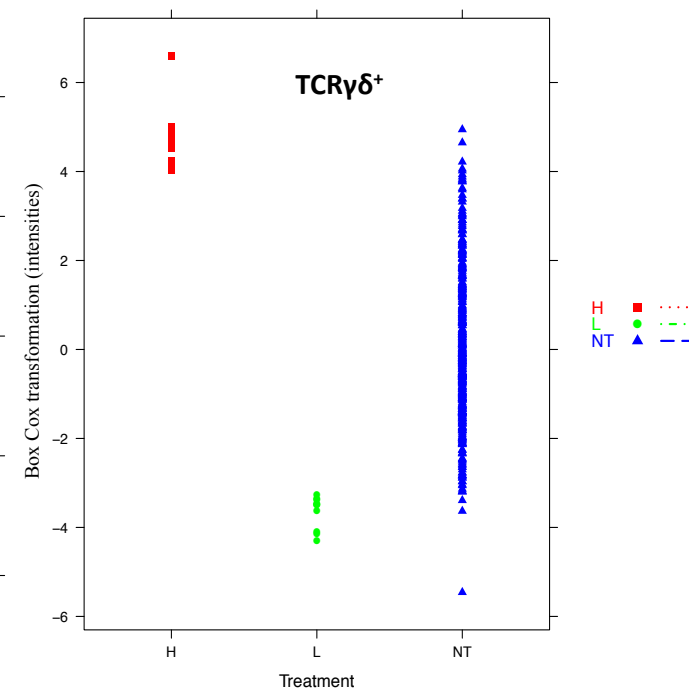

Supplement: Additional file 2: Figure S2 — Distribution of the individual’s values for in vivo immune traits. The values of animals from the high and low groups are labelled by colour: green “Low” and red “High”. [file 1471-2164-14-894-S2.pdf]

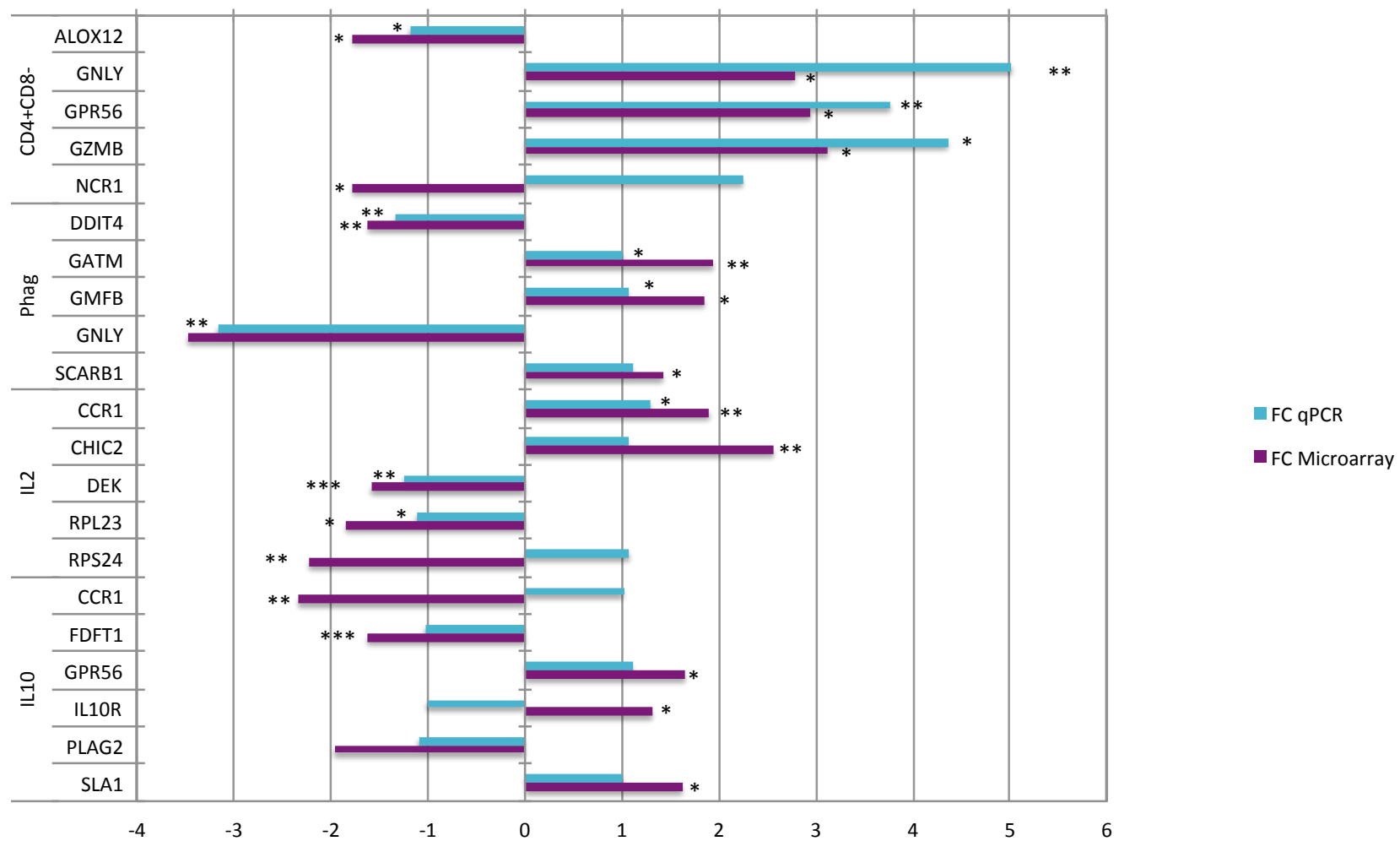

Supplement: Additional file 4: Figure S3 — Validation of the transcriptome results by RT-qPCR on a subset of genes. [file 1471-2164-14-894-S4.pdf]

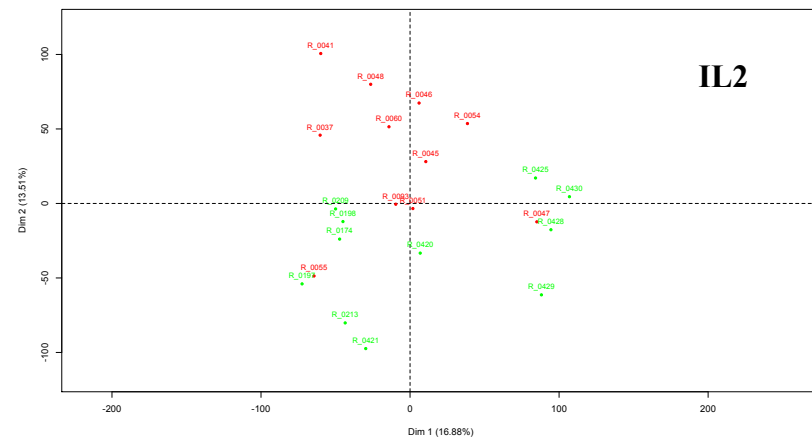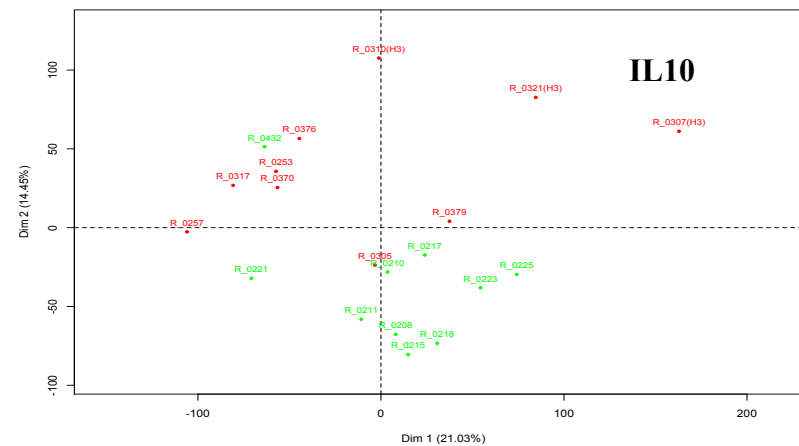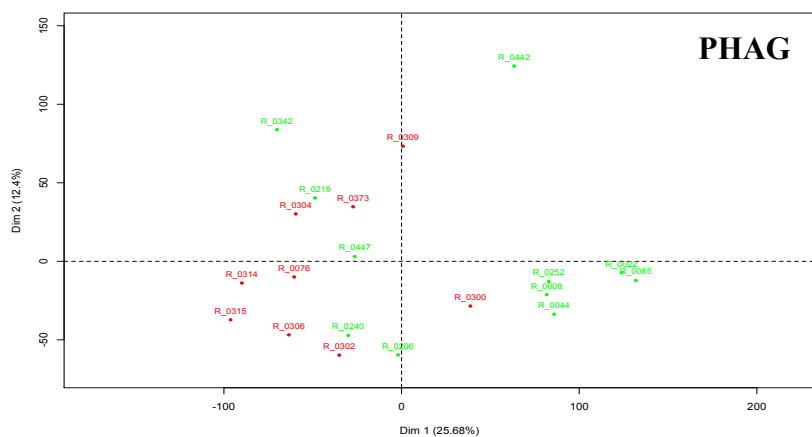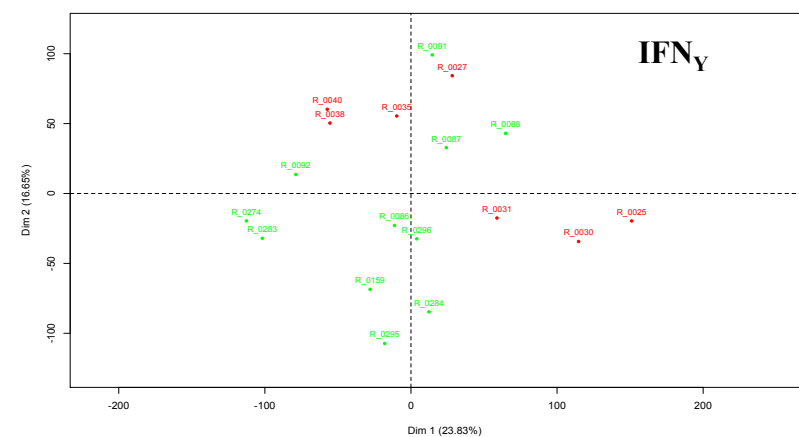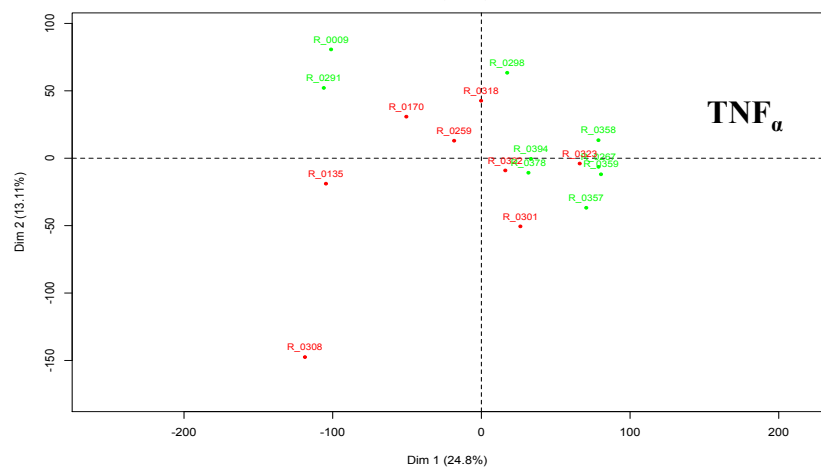

Supplement: Additional file 14: Figure S4 — Principal component analysis (PCA) of microarray expression data for in vitro immune traits. PCA was performed with FactoMineR library (version 1.23) to detect if any particular array largely contributed to variability in the gene expression data, that is, retains most information. Animals are labelled by colour: green “Low”; and red “High”. [file 1471-2164-14-894-S14.pdf]

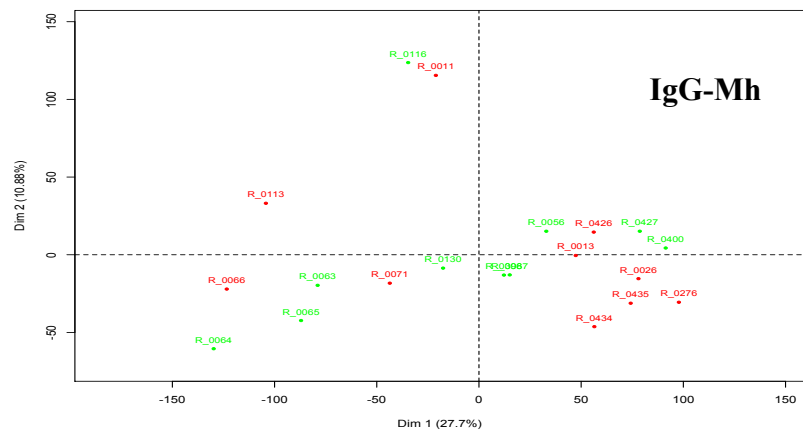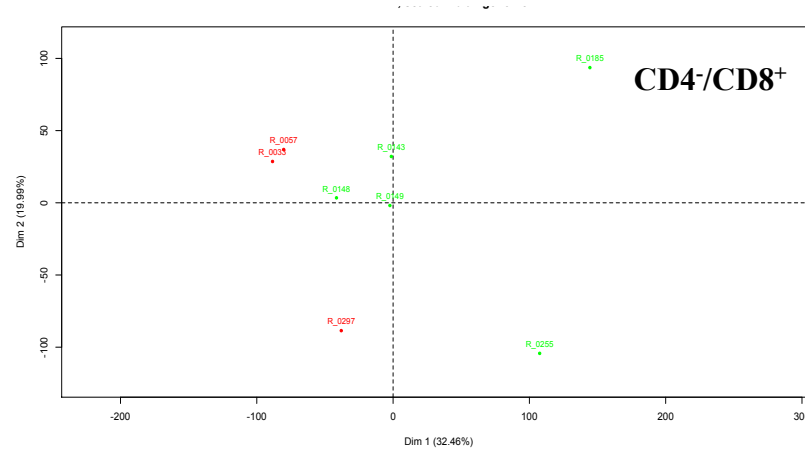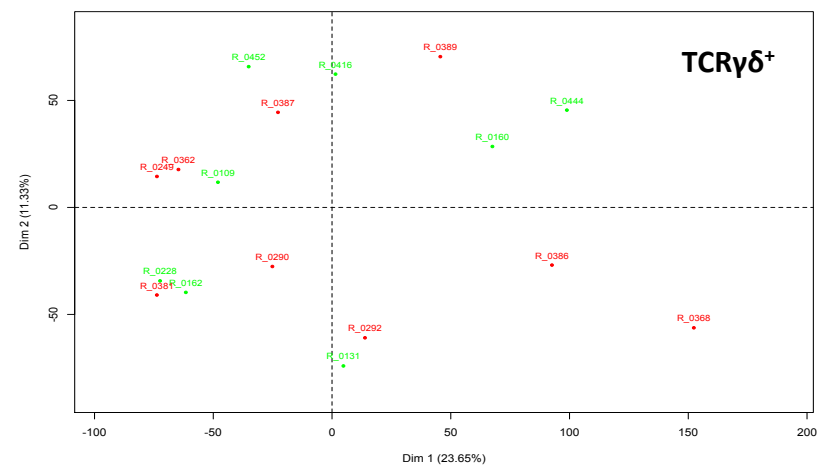

Supplement: Additional file 15: Figure S5 — Principal component analysis (PCA) of microarray expression data for in vivo immune traits. PCA was performed with FactoMineR library (version 1.23) to detect if any particular array largely contributed to variability in the gene expression data, that is, retains most information. Animals are labelled by colour: green “Low”; and red “High”. [file 1471-2164-14-894-S15.pdf]
