# Supplementary material for: The peripheral blood transcriptome reflects variations in immunity traits in swine: towards the identification of biomarkers
Source: BMC Genomics. 2013 Dec 17;14:894. doi: 10.1186/1471-2164-14-894 (PMC3878494; doi:10.1186/1471-2164-14-894)
Supplement: Additional file 3 — Sequences of the primers used for the RT-qPCR. [file 1471-2164-14-894-S3.docx]

### Additional file 1 – Sequences of the primers used for quantitative real time PCR

In order to validate the microarray results, a set of genes were analyzed by RT-qPCR. The genes were selected based on the following strategies: (1) genes with significant differential expression levels between the H and L groups for a target IT that spanned a dynamic range of at least log2 (ratio) > 0.485; (2) genes with a coefficient of determination greater than 0.8 with respect to the first principal component in the PCA; and (3) genes with biological interest (e.g. swine leukocyte antigen 1 (*SLA-1*) and IL10 receptor (*IL10RA*))

| Type of traits | Trait symbol | *Gene* | Sequences | |
| --- | --- | --- | --- | --- |
|  |  |  | Forward | Reverse |
|  |  |  |  |  |
| *In vitro* | IL2 | *CCR1* | 5’-TGGCTCTCCCAACCAGATTC-3’ | 5’-ACCCCACGTGGCTACAATTC-3’ |
|  |  | *CHIC2* | 5’-GGTCACGTCACCGTATTTGGA-3’ | 5’-CCAGTTAAAGAAGAAGGGAATTCTGA-3’ |
|  |  | *DEK* | 5’-AAGTAGAGAGGTTGACAATGCAAGTC-3’ | 5’-TCCCTTCCCCTGTGCAATT-3’ |
|  |  | *RPL23* | 5’-GCAGACTTGTGGCCCAGAA-3’ | 5’-CACTGAAGAATCACGCAATGCT-3’ |
|  |  | *RPS24* | 5’-CGCCATCATGAACGACACA-3’ | 5’-TGAAGTAGTCGGTTGGTCATGAA-3’ |
|  | IL10 | *CCR1* | 5’-TGGCTCTCCCAACCAGATTC-3’ | 5’-ACCCCACGTGGCTACAATTC-3’ |
|  |  | *FDFT1* | 5’-TGAATCAGACCAGTCGCAGTTT-3’ | 5’-GCGCATTTCCCCATCCA-3’ |
|  |  | *GPR56* | 5’-GACCACAGCTGGCAGGTTAAA-3’ | 5’-CAGCCGAACCGCCTTTC-3’ |
|  |  | *IL10R* | 5’-GGGCCGTGGACAAAAGC-3’ | 5’-GTCAACCGTCAGAGTCACTTCATC-3’ |
|  |  | *PLAGL2* | 5’-CCCCCATCCAGAGCAGAGA-3’ | 5’-GGGTGGCCATGTGCCTATAC-3’ |
|  |  | *SLA-1* | 5’-TCCGAGTGAACCTGAACAACCT-3’ | 5’-CAGCCGTACATGCTCTGGAA-3’ |
|  | PHAG | *DDIT4* | 5’-GGCTGTACGCACTCGTCAGA-3’ | 5’-AGCGCAGAAGACGCAGCTA-3’ |
|  |  | *GATM* | 5’-AGGTGAAGGCCAACACATATGA-3’ | 5’-GGCAACAGCCTTTTTCAAATG-3’ |
|  |  | *GMFB* | 5’-ACGACAACCTCGCTTCATTGT-3’ | 5’GAAAATAAAGCACAGAGGGTATGAAAC-3’ |
|  |  | *SCARB1* | 5’-TGGGCGCTGTTATGATCGT-3’ | 5’-TCGATGCGCACATTCTTGAG-3’ |
|  |  | *GNLY* | 5’-ATGCGACGGAGAGCAGTTCT-3’ | 5’-TTCCGACAAGACTCACAGATGAG-3’ |
| *In vivo* | CD4^-^/CD8^+^ | *ALOX12* | 5’-GGCAAGGAGACGGAATTCAGT-3’ | 5’-CCTGAAGGAGGTGCCGTTT-3’ |
|  |  | *GNLY* | 5’-ATGCGACGGAGAGCAGTTCT-3’ | 5’-TTCCGACAAGACTCACAGATGAG-3’ |
|  |  | *GPR56* | 5’-GACCACAGCTGGCAGGTTAAA-3’ | 5’-CAGCCGAACCGCCTTTC-3’ |
|  |  | *GZMB* | 5’-ACGAGAAGCGCATCTCCAAT-3’ | 5’-GAGGGTCTTCACAGCCTTAGTCA-3’ |
|  |  | *NCR1* | 5’-AGCAGCAGACCCTCTCCAAAC-3’ | 5’-TCGAAGAGCAGCTGGTACTCAA-3’ |
|  |  |  |  |  |
|  |  | *RPL32^1^* | 5’-TGCTCTCAGACCCCTTGTGAAG-3’ | 5’-TTTCCGCCAGTTCCGCTTA-3’ |
|  |  | *B2M^1^* | 5’-TGGTCTTTCTACCTTCTGGTCC-3’ | 5’-TGTGATGCCGGTTAGTGGTCTC-3’ |

^1^Reference genes
